# The Effectiveness of Self-Management Strategies in Patients With Heart Failure: A Narrative Review

**DOI:** 10.7759/cureus.41863

**Published:** 2023-07-14

**Authors:** Josephine Koikai, Zahid Khan

**Affiliations:** 1 Internal Medicine, Kenyatta National Hospital/ University of Nairobi (KNH/UoN), Nairobi, KEN; 2 Acute Medicine, Mid and South Essex NHS Foundation Trust, Southend-on-Sea, GBR; 3 Cardiology, Barts Heart Centre, London, GBR; 4 Cardiology and General Medicine, Barking, Havering and Redbridge University Hospitals NHS Trust, London, GBR; 5 Cardiology, Royal Free Hospital, London, GBR

**Keywords:** heart failure self management care, intervention reduced hospitalization, hospitalization for heart failure, lite and plus programs, individualized education intervention, self management strategies, acute decompensated heart failure, heart failure with preserved ejection fraction, echocardiography - heart failure - valvular heart disease, heart failure with reduced ejection fraction

## Abstract

Heart failure (HF) is a common condition with high morbidity and mortality. Self-management strategies for heart failure can be effective in improving patients' quality of life and reducing mortality and hospitalization for heart failure. These self-management strategies are also cost-effective. A complex interplay between various factors related to patients, therapy, healthcare, and socioeconomic factors influences the effectiveness of self-management strategies. The primary aim of this study is to determine the effectiveness of self-management strategies in patients with heart failure in reducing mortality, hospitalization for heart failure, and healthcare cost savings at six months and one year. The secondary aim is to determine adherence to self-management strategies in patients with HF.

The current study is a narrative review of studies evaluating the effectiveness of self-management strategies in heart failure. A literature search was done in PubMed, Embase, Google Scholar, ScienceDirect, and the Cochrane Library for studies published in the English language between 2012 and 2022. Descriptive statistics were used to summarize the characteristics of studies and interventions. We calculated odds ratios, risk ratios, or mean differences to calculate the effect of self-management strategies on mortality, hospitalization for HF, and healthcare costs between patient groups. We included a total of 30 studies in our narrative review: eight cross-sectional studies and 22 randomized controlled trials.

These studies showed a significant effect of self-management strategies on mortality at six- and 12-month follow-ups. Studies on the effectiveness of self-management strategies on hospitalization for heart failure showed benefits at six and 12 months. Self-management strategies are cost-effective and feasible with improved disability-adjusted life years (DALY). One study showed higher costs associated with self-management strategies and only a slight decrease in DALY. Overall, adherence to self-management strategies was inadequate in these studies. Novel and innovative self-management interventions improve therapy adherence. There was a lack of uniformity in using tools to assess self-management across studies. There was a lack of ethnic diversity in the individual studies, limiting the generalization of these studies' findings.

Our review showed that self-management strategies are beneficial for heart failure-related hospitalization, reduce mortality and hospitalization for heart failure, and are cost-effective. The use of innovative approaches like smartphone applications improves adherence.

## Introduction and background

Heart failure (HF) is a clinical syndrome characterized by typical symptoms and signs resulting from a structural or functional heart abnormality [1]. It is a common condition with high morbidity and mortality. According to the 2017 Burden of Heart Failure data, the age-standardized prevalence rate of HF in Europe is 1,058.1/100,000, 960.4/100,000 in the United States of America (USA), 700/100,000 in Eastern Sub-Saharan Africa, and 700.7/100,000 in Kenya [2]. In Europe, the one-year combined all-cause mortality and hospitalization rate for heart failure is 36% [3]. In comparison, the one-year mortality rate and hospitalization rate for HF in the USA are 37.5% and 30.9%, respectively [4]. The one-year all-cause mortality rate is 34% for Sub-Saharan Africa [5]. A study at a tertiary care hospital in Kenya reported a four- to six-month hospitalization rate for HF and a mortality rate of 38% and 25%-38%, respectively [6].

Self-management

Self-management is the act of sustaining health through health promotion and prevention practices [7]. It is one of the core components of an HF management program [1]. The three main successful self-management strategies in HF include self-care maintenance, self-care monitoring, and self-care management. Self-care maintenance involves adhering to practices and behaviors to maintain physical and emotional stability. Neuro-humoral activation and systemic inflammation play a role in the pathophysiology of HF. Some general health and HF-specific self-care maintenance behaviors like physical activity result in partial neurohumoral deactivation and a reduction in biomarkers of systemic inflammation [8,9]. Self-care monitoring involves self-recognition of heart failure-attributed changes, such as a change in weight.

Self-care management is the action taken in response to a change in symptoms or signs of HF, like the titration of diuretics with the development of edema. Peripheral edema and clinical congestion are the predominant reasons for heart failure-related hospitalization [10]. The greatest efficacy of HF medications occurs in the absence of clinical congestion [11]. Patients who recognize and manage edema effectively get optimal benefits from their HF medication. Emphasis has been laid on several aspects of self-management behavior, including activity and exercise, sleep and breathing, restricted fluid intake, a healthy diet, immunization, alcohol, smoking, and recreational drug cessation, travel, leisure, and driving, sexual activity, and symptom self-management [1].

A meta-analysis of 20 randomized controlled trials (RCTs) based on 5,624 patients demonstrated a reduction in the risk of HF-related hospitalization and mortality by 1%-4% for each increasing month when self-management intervention was undertaken [12]. The European Society of Cardiology gives a class 1A recommendation for the adoption of self-management strategies in patients with HF to reduce the risk of hospitalization and mortality [1]. In addition, these strategies are also cost-effective. In a randomized controlled trial (RCT) of 223 patients with HF, the cost of care for patients randomized to the intervention, i.e., education on self-management, was lower by $2,823 per patient compared to the control group after factoring in the cost of the intervention [13]. In another RCT of 190 patients with HF, there was a mean annual reduction in the cost of care of $1,300 per patient in those who were randomized to self-management [14].

Adherence

Adherence to long-term therapies has become an emerging issue. The World Health Organization (WHO) defines adherence as ‘the extent to which a person’s behavior corresponds with agreed-upon recommendations from a health care provider’ [15]. Factors related to health care, socioeconomic, therapy, patient, and condition-related factors influence the degree to which a patient is adherent to prescribed therapy [15].

Barriers in either of these five domains may lead to suboptimal adherence to self-management strategies in HF. Interventions targeting more than one of these domains should be put in place to ensure optimal adherence to self-management strategies and achieve a reduction in heart failure-related hospitalization, mortality, and cost savings. In a study on heart failure self-management strategies at a tertiary care hospital in Kenya, 50.8% of patients with HF had poor self-care practices [16].

## Review

Heart failure is a common condition with high morbidity and mortality. Self-management strategies in HF, besides being cost-effective, are also effective in improving the health-related quality of life of patients and reducing hospitalization for heart failure and mortality [1,13-21]. The primary aim of this study is to determine the effectiveness of self-management strategies in reducing mortality and morbidity in patients with heart failure. In addition, this study also aims to assess the healthcare cost savings after six months and one year of adopting these strategies. The secondary aim is to determine the adherence to self-management strategies of patients with HF.

Methodology

Study Design

This study is a narrative review of studies evaluating the effectiveness of self-management strategies in heart failure. Fidelity to the Preferred Reporting Items for Systematic Reviews and Meta-analyses (PRISMA) guidelines was observed.

Search Strategy

A literature search was done in several databases, including PubMed, Embase, Google Scholar, ScienceDirect, and the Cochrane Library, for published literature over the last 10 years based on the inclusion and exclusion criteria discussed below. Medical Subject Heading (MeSH) words used for the search were "self-maintenance", "self-monitoring", "self-management", "lifestyle", "physical activity", "fluid and diet", "smoking, alcohol, recreational drugs", "sexual activity, travel, leisure, driving", "immunization", "symptom self-management", "sleep hygiene" and "congestive heart failure", cardiac failure", "myocardial failure", "heart failure with reduced ejection fraction", "heart failure with mildly reduced ejection fraction", "heart failure with preserved ejection fraction", "left-sided heart failure" (Table 1).

**Table 1:** MeSH terms used in the literature search MeSH: Medical Subject Heading

Self-management strategies	Components of self-care	Heart failure
Self-maintenance, self-monitoring, self-management, self-care	Lifestyle, physical activity, fluid intake and diet, smoking, alcohol, recreational drugs, sexual activity, travel, leisure, and driving, immunization symptoms, self-management, sleep hygiene	Congestive heart failure, cardiac failure, myocardial failure heart failure with reduced ejection fraction, heart failure with mildly reduced ejection fraction, heart failure with preserved ejection fraction, left-sided heart failure

Study Selection

Following the literature search, duplicate articles were removed by using the Mendeley citation manager. Two reviewers screened the articles by title and abstract to filter out articles unrelated to the study. The full text of the remaining articles was reviewed for inclusion and exclusion from the study.

Eligibility criteria

Inclusion Criteria

The inclusion criteria were: i. Observational, randomized, and non-randomized control studies published in English between 2012 and 2022; ii. Studies with participants who were 18 years old or older; iii. Quantitative results were reported; iv. Studies that report on at least half of the aspects of self-management behavior; v. Patients with heart failure with reduced ejection fraction (HFrEF), mildly reduced ejection fraction, and heart failure with preserved ejection fraction (HFpEF).

Exclusion Criteria

Case reports case series, editorials, and review articles were excluded.

Data management and quality assessment

Summary data were tabulated and extracted onto Microsoft Excel (Microsoft Corp., Redmond, Washington, United States) forms, and IBM Statistical Package for the Social Sciences (SPSS) (IBM Corp., Armonk, New York, United States) software was used for data analysis. Two reviewers assessed the methodological quality of the included studies using the Delphi list for randomized controlled studies and the Modified Newcastle-Ottawa tool for observational studies. Descriptive statistics were used to summarize the characteristics of studies, samples, and interventions. Continuous data were summarized by means and standard deviations, and categorical data were summarized by frequencies and percentages. Between-group estimates that quantify the effect of self-management strategies on mortality, hospitalization for HF, and healthcare costs were summarized by risk ratios, odds ratios, or mean differences. Adherence was summarized as a proportion of those adhering to self-management strategies.

Results

**Figure 1:**
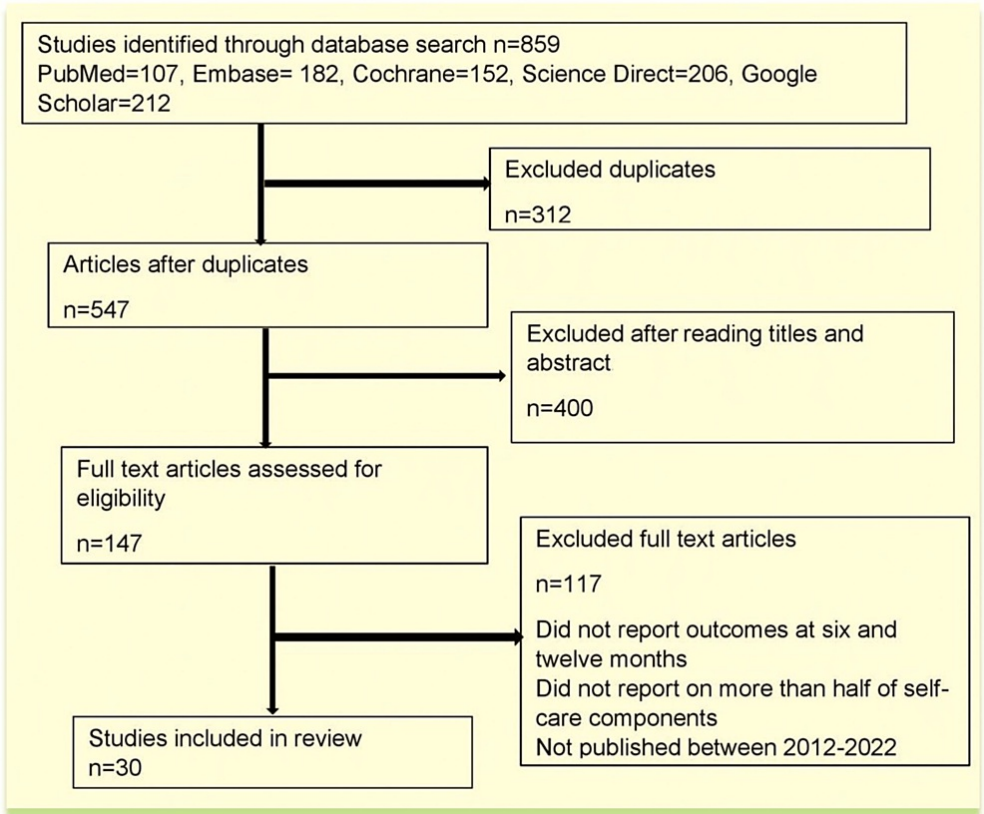
Study flow diagram

Most studies on the effectiveness of self-management strategies on clinical outcomes in patients with heart failure have included other interventions in addition to self-management. This makes it difficult to isolate the impact of self-management strategies alone among these other interventions. However, some studies have focused on the effect of self-management strategies on reducing mortality, hospitalization for heart failure, cost-effectiveness, and adherence practices. The database search in PubMed, Cochrane Library, Science Direct, Embase, and Google Scholar using the MeSH terms specified resulted in 859 studies that were screened for duplicates through the Mendeley citation manager, and 312 studies were removed as a result. We screened the remaining 547 studies by reading their titles and abstracts, resulting in the exclusion of 400 studies for not meeting the inclusion criteria. The remaining 147 studies were assessed for eligibility for inclusion in the review, and another 117 studies were excluded as they did not report outcomes at six or 12 months, did not report on more than half of the components of self-care, or were published before 2012. Only 30 articles met the inclusion criteria, and the characteristics of these studies are presented in Figure 1.

We did not find any studies from the African continent reporting on the effectiveness of self-management strategies on mortality in patients with heart failure. The included studies were equivocal in reporting the effect on mortality, with all showing a significant benefit except one study by Kessing et al. [22]. This study compared patients with high versus low self-care and mortality. Self-care was assessed using the European Heart Failure Self-Care Behavior Scale, and a median split was used to determine those who had high self-care versus those with low self-care since the score does not provide an interpretation for this. This could have led to the wrong stratification of the participants and thus insignificant results. Bekelman et al. only reported the number needed to treat the analysis [21] (Tables 2-3).

**Table 2:** Characteristics of studies on the effectiveness of self-management strategies on mortality RCT: randomized controlled trial; OR: odds ratio; CI: confidence interval; HR: hazard ratio; HFrEF: heart failure with reduced ejection fraction; HFpEF: heart failure with preserved ejection fraction

Study	Design	Year	Setting	Patients	Intervention	Comparison	Outcome
							Mortality
Deek et al. [18]	RCT	2020	Lebanon	n=218, mean left ventricle ejection fraction (LVEF): 36%, (SD 12)	A single educational session in self-care, self-care materials for patients and carers	Self-care material only	Reduction in mortality in the intervention group vs control group at six months (OR=2.5, 95% CI, 1.35-4.76, p=0,02) and at 12 months (OR=2, 95% CI, 1.11-3.33, p=0.01)
Smith et al. [19]	RCT	2014	USA	n=198, mean LVEF: overall 30%	Four weekly group visit appointments, education on components of self-care	Usual care	More deaths in the control group (28%) versus the intervention group (24%) HR=0.45, 0.21-0.98, 95% CI
Hwang, Huh, Jeong, Cho, & Lee. [20]	RCT	2022	South Korea	n=122, mean LVEF: 40.3% ± 14.23	Individualized education intervention	Usual care	Mortality 12% in intervention, 24% in control adjusted HR=0.40, 95% CI, 0.16-0.98, p=0.046
Bekelman et al. [21]	RCT	2015	USA	n=392 included normal, preserved, mildly reduced, and reduced LVEF; majority normal	Patient-centered disease management	Usual care	4.3% mortality in the intervention group versus 9.6% in the control group P=0.04; Number needed to treat 20, 95% CI, 10-307
Kessing, Denollet, Widdershoven, & Kupper [22]	Cohort	2016	Netherlands	n=559, LVEF: 31.7%±7.1	High global self-care score	Low global self-care score	No significant benefit in mortality
Nakane et al. [23]	Cohort	2021	Japan	n=569, LVEF mean: 42.7%	Self-care management system	Usual care	Composite of all-cause death and hospitalization (24.5% versus 34.9% p=0.031; HR 0.62, 95% CI, 0.40-0.96)
Dracup et al. [24]	RCT	2014	USA	n=614, HFrEF (51%) and HFpEF (49%)	LITE and PLUS programs	Usual care	Reduced cardiac death in LITE compared to control P=0.003

**Table 3:** Characteristics of studies on the effectiveness of self-management strategies on hospitalization for heart failure RCT: randomized controlled trial; LVEF: left ventricle ejection fraction; OR: odds ratio; RR: risk ratio; CI: confidence interval; HOM-HEMP: home-based heart failure self-management program; ICALOR: Insuffisance CARdiaque en Lorraine; REACH-HFpEF: Rehabilitation EnAblement in Chronic Heart Failure; HF S2S: heart failure self-care program Self-care to Success

Study	Design	Year	Setting	Patients	Intervention	Comparison	Outcome
							Hospitalization for heart failure
Boyde et al. [25]	RCT	2018	Australia	n=200 included normal, preserved, mildly reduced, and reduced LVEF	Educational intervention	Usual care	The intervention reduced the risk of hospitalization by 30% (OR=0.407, 95% CI, 0.216-0.766)
Chi & Chen [26]	RCT	2012	China	n=171 included normal, preserved, mildly reduced, and reduced LVEF	Daily-based self-management intervention	Usual care	The intervention reduced hospitalization. RR=0.41, 95% CI, 0.21-0.79
Xiaoniong et al. [27]	RCT		China	n=96, LVEF 42.1%±2.3	Structured education	Usual care	Lower readmission in intervention (10.4%) versus control (27.1%) p=0.036
Mizukawa et al. [28]	RCT	2019	Japan	n=59 , LVEF 42.1±16.5	Self-management and collaborative management	Usual care	Lower hospitalization in the intervention groups versus the usual care group
Jiang et al. [29]	RCT	2021	Singapore	n=213, LVEF not specified	HOM-HEMP intervention	Usual care	Reduced hospitalization in the intervention versus control group, p=0.016
Cockayneet al 2014. [30]	RCT	2014	United Kingdom	n=260, LVEF not specified	Nurse facilitated self-management	Self-facilitated management	Intervention group less likely to be admitted compared to control (19% and 21.2% respectively, p=0.66 *did not reach statistical significance
Agrinier et al. [31]	Observational cohort	2013	France	n=1223, mean LVEF 35%	ICALOR	-	183 readmissions averted (7.19%)
Sezgin et al. [32]	RCT	2017	Turkey	n=90, mean LVEF 30.22%	Self–care program	Usual care	No difference at six months
Lang et al. [33]	RCT	2018	Scotland	n=50, LVEF >45%	REACH-HFpEF	Usual care	Four hospitalizations in the intervention vs seven in the control group
Bryant & Himawan. [34]	Pre-post interventional	2019	USA	n=67, LVEF not specified	HF S2S	-	Drop in hospitalization to 0.175, s=0.446

All studies except Sezgin et al. reported a benefit of the self-management intervention in reducing hospitalization for heart failure. Sezgin et al. [32] reported no significant benefit between the intervention and usual care groups at six months. This could be due to the shorter follow-up duration and the smaller number of randomized patients in the study. There was no blinding of treatment allocation done by Lang et al., and there was a significant difference in the baseline characteristics of study participants [33].

Four studies on cost-effectiveness in this review showed the cost-effectiveness of self-management interventions without any negative influence on the disability-adjusted life years or the quality-adjusted life years, except Mejía et al., who reported an increase in the cost of the cognitive behavior-based self-care intervention with a slight reduction in disability-adjusted life years [36] (Table 4).

**Table 4:** Characteristics of studies on the cost-effectiveness of self-management strategies LVEF: left ventricle ejection fraction; REACH-HF: Rehabilitation EnAblement in CHronic Heart Failure; DALY: disability-adjusted life years

Study	Design	Year	Setting	Patients	Intervention	Comparison	Outcome
							Cost-effectiveness
Dalal et al. [35]	RCT	2019	United Kingdom	n=216, LVEF median: 34.5 (25-39)	REACH-HF, a novel home-based program	Usual care	£418.39 cost of intervention per patient, feasible
Mejía, Richardson, Pattenden, Cockayne, & Lewin [36]	RCT	2014	United Kingdom	n=260, LVEF not specified	Nurse facilitated self-management program. Six one on one education sessions with a nurse	Self-management program followed by oneself	The increased cost of intervention by £69.49, reduction in DALY by 0.004
Maru et al. [37]	RCT	2015	Australia	n=280, LVEF: 36% ±14.2	Home-based intervention	Clinic-based intervention	A home-based intervention was more cost-effective
Reilly et al. [38]	RCT	2015	USA	n=134, LVEF=33.9 ±17.6	Education on self-care, home visits, follow-up telephone calls	Usual care	Health resource use was lower in the intervention group (£9065) vs control (£16712) with stable DALY

A limited number of observational studies have been done in Africa, especially in Ethiopia and South Africa, assessing the adherence to self-care in heart failure and the use of novel approaches to improve adherence to self-care, such as telemonitoring and the use of smartphone applications. All studies included in this review showed poor adherence to key components of self-management strategies such as weight monitoring, fluid intake, and diet. The tools used to assess adherence, although validated, were not uniform across all studies, with some studies using the Self-Care in Heart Failure Index and others using the European Heart Failure Self-Care Behavior Scale (Table 5).

**Table 5:** Characteristics of studies on adherence to self-management strategies RCT: randomized controlled trial; LVEF: left ventricle ejection fraction; ITEC-CHF: innovative telemonitoring enhanced care program for chronic heart failure; SCHFI: Self Care for Heart Failure Index; PATCH: Patient AcTivated Care at Home

Study	Design	Year	Setting	Patients	Intervention	Comparison	Outcome
							Adherence
Dessie et al. [39]	RCT	2021	Ethiopia	n=219, LVEF not specified. Stage four heart failure: 77%	Self-care education in hospital post-discharge and follow-up	Usual care	Improved mean self-care adherence score from baseline 12.6(±2.5) to 20.9(±7.04) vs 12.1(±2.8) to 12.6(±2,5)
Baymot, Gela, & Bedada [40]	Cross-sectional	2022	Ethiopia	n=294, LVEF 38% ±14.7	-	-	32.7% good adherence
Fetensa et al. [41]	Cross-sectional	2021	Ethiopia	n=424, LVEF not specified	-`	-	51.2% good adherence
Seid, Abdela, & Zeleke [42]	Cross-sectional	2019	Ethiopia	n=310, LVEF not specified	-	-	22.3% good adherence
Verena et al. [43]	Cross-sectional	2012	South Africa	n=200, LVEF 32±8	-	-	2.5-98% adherence to self-care components
Ding et al. [44]	RCT	2020	Australia	n=184, 29.4% (6.5)	ITEC-CHF program, usual care	Usual care	Improved self-adherence (health maintenance, medication, and diet) score from baseline with intervention at six months
Chew, Sim, Choi, & Chair [45]	RCT	2021	Singapore	n=144, LVEF 33.7%±12.5	Face-to-face sessions, printed heart failure manual, reinforcement calla	Usual care	Poor adherence to self-care at baseline SCHFI 52.9±17.2
Young, Hertzog, & Barnason [46]	RCT	2019	USA	n=100, LVEF 55.7%±11.1	PATCH intervention	Usual care	Higher self-reported adherence score on self-care components (exercise, weight checks, low salt diet)
Janssen-Boyne et al. [47]	RCT	2014	Netherlands	n=382, mean LVEF 38%	Healthy Buddy	Usual care	Daily compliance to self-care via Healthy Buddy was 90%

Risk of bias assessment

Randomized controlled trials were assessed using the nine-point Delphi list. The questions included in the list are:

Q1a. Was a method of randomization applied? Yes/No/Don’t know; Q1b. Was the treatment allocation concealed? Yes/No/Don’t know.

Q2. Were the groups similar at baseline regarding the most important prognostic indicators? Yes/No/Don’t know.

Q3. Were the eligibility criteria specified? Yes/No/Don’t know.

Q4. Was the outcome assessor blinded? Yes/No/Don’t know.

Q5. Was the care provider blinded? Yes/No/Don’t know.

Q6. Was the patient blinded? Yes/No/Don’t know.

Q7. Were point estimates and measures of variability presented for the primary outcome measures? Yes/No/Don’t know.

Q8. Did the analysis include an intention-to-treat analysis? Yes/No/Don’t know.

Table 6 summarizes the risk of bias assessment for randomized controlled studies included in the review.

**Table 6:** Risk of bias assessment for included randomized controlled studies Q: question; Y-yes; N-no

Study	Q1a	Q1b	Q2	Q3	Q4	Q5	Q6	Q7	Q8
Deek et al. [18]	Y	Y	Y	Y	Y	Y	Y	Y	N
Smith et al. [19]	Y	Y	Y	Y	Y	Y	Y	Y	N
Hwang et al. [20]	Y	Y	Y	Y	Y	Y	Y	Y	N
Bekelman et al. [21]	Y	Y	Y	Y	Y	Y	Y	Y	Y
Boyde et al. [25]	Y	Y	Y	Y	Y	Y	Y	Y	N
Chi & Chen [26]	Y	Y	Y	Y	Y	Y	Y	Y	N
Xiaoniong et al. [27]	Y	N	Y	Y	Y	Y	Y	Y	N
Mizukawa et al. [28]	Y	Y	Y	Y	Y	Y	Y	Y	N
Jiang et al. [29]	Y	Y	Y	Y	Y	Y	Y	Y	N
Cockayne et al. [30]	Y	Y	Y	Y	Y	Y	Y	Y	N
Dalal et al. [35]	Y	Y	Y	Y	Y	Y	Y	Y	N
Mejía et al. [36]	Y	N	Y	Y	Y	Y	Y	Y	N
Maru et al. [37]	Y	Y	Y	Y	Y	Y	Y	Y	N
Reilly et al. [38]	Y	Y	Y	Y	Y	Y	Y	Y	N
Dessie et al. [39]	Y	N	Y	Y	Y	Y	Y	Y	N
Ding et al. [44]	Y	Y	Y	Y	Y	Y	Y	Y	N
Chew et al. [45]	Y	Y	Y	Y	Y	Y	Y	Y	N
Young et al. [46]	Y	Y	Y	Y	Y	Y	Y	Y	N
Sezgin et al. [32]	Y	Y	Y	Y	Y	Y	Y	Y	N
Lang et al. [33]	Y	N	N	N	N	N	N	N	N
Janssen-Boyne et al. [47]	Y	Y	Y	Y	Y	Y	Y	Y	N
Dracup et al. [24]	Y	Y	Y	Y	Y	Y	Y	Y	N

The Modified Newcastle-Ottawa Score was used in the risk of bias assessment for observational studies. The three domains in this score include selection, comparability, and the outcome of observational studies. Table 7 provides a summary of the risk of bias assessment for observational studies.

**Table 7:** Risk of bias assessment for the included observational studies

Study	Selection	Comparability	Outcome
Baymot et al. [40]	4	-	2
Fetensa et al. [41]	3	-	2
Seid et al. [42]	3	-	2
Verena et al. [43]	4	-	2
Kessing et al. [22]	3	-	2
Nakane et al. [23]	3	-	3
Agrinier et al. [31]	4	-	2
Bryant et al. [34]	4	-	2

Discussion

Effectiveness of Self-Management Strategies on Mortality

Deek et al. performed an extended follow-up multi-site randomized controlled trial at three tertiary medical centers in Beirut to evaluate the impact of a single educational intervention on heart failure self-care [18]. Two hundred and eighteen patients were randomized to the intervention (n=113) and control (n=103) groups, respectively. The mean age of patients was 67 years, and the majority were male (59%). The mean left ventricle ejection fraction (LVEF) was 36%. At six months, mortality in the intervention group was 16% compared to 33% in the control group (odds ratio (OR)=2.5, 95% confidence interval (CI), 1.35-4.76, p=0.02). At 12 months, mortality in the intervention group was 26% versus 42% in the control group (OR=2, 95% CI, 1.11-3.33, p=0.01). Forty-three percent of participants were readmitted to the hospital at six months and 53% at 12 months, with no significant differences between the intervention and control groups.

The Self-Management and Care of Heart Failure (SMAC-HF) trial involving Medicare patients evaluated the effectiveness of multidisciplinary group clinic appointments in educating patients with heart failure on self-management skills [19]. One hundred and ninety-eight patients hospitalized with heart failure were randomized to the intervention (n = 92) or standard care group (n = 106) and followed up for 12 months. The mean age was 62.3 years, and women made up only 38% of the study participants. The mean LVEF was 30%, with only 7% of patients having an LVEF > 40%. The primary outcome occurred in 24% of the participants in the intervention group compared to 28% in the standard care group (hazard ratio (HR)=0.45, 0.21-0.98, 95% CI). Hwang et al. [20] conducted a randomized controlled trial on hospitalized patients with heart failure at a university hospital in Seoul, intending to examine the effects of an educational intervention on patient-reported outcomes and all-cause mortality. One hundred and twenty-two patients were enrolled, with 60 randomized to the intervention group and 62 to the control group. The average age of the participants was 66.22 years, and 50.8% were female. The mean LVEF was 40.3%, with 51.6% having an LVEF <40%. The mortality rate was 12% in the intervention group compared to 24% in the control group (adjusted HR=0.40, 95% CI, 0.16-0.98, p=0.046)

Bekelman et al. [21], in a multicenter randomized controlled trial, sought to determine the effectiveness of a collaborative care patient-centered disease management (PCDM) intervention to improve the health status of patients with heart failure. One component of the PCDM was telemonitoring with self-management support. The other two components were multidisciplinary collaborative care for heart failure disease management and screening and treatment of depression. Three hundred and ninety-two patients were enrolled from multiple Veteran Affairs centers in North America. The mean age did not differ at baseline for both groups (67.3 years in the intervention group, 67.9 years in the control group). Male participants were significantly more than females in both groups (95.2% and 98%), considering this is a veteran affairs population. Most patients had normal left ventricle ejection fractions in both groups (45.6% in the intervention group, 47.5% in the control group). Patients were randomized to usual care (n=199) or usual care plus PCDM intervention (n=193); 4.3% of patients in the PCDM intervention arm died compared to 9.6% in the usual care arm p=0.04. The number needed to treat was 20 (95% CI, 10-307). No significant difference was noted between the usual care and intervention groups regarding hospitalization at one year (29.9% and 29.4%, respectively, p=0.87). The other secondary outcome was depression, measured by the Patient Health Questionnaire 9 (PHQ9). Kessing et al. [22] conducted a cohort study on the association of self-care with all-cause mortality in 559 patients with heart failure from three health facilities in the Netherlands. The mean age was 66.3 years in both groups; male participants were more in number (75%). The average LVEF was 31.7% ±7.1. There was no significant benefit in mortality for patients with high versus low scores in global self-care. Interestingly, after conducting a post hoc analysis, low self-reported sodium intake was associated with increased all-cause mortality after adjusting for demographic and clinical factors (HR=1.47,95% CI, 1.10-1.97, p=0.01).

Nakane et al. [23] enrolled 569 patients retrospectively from a health facility in Japan and prospectively followed them up, with 275 patients in the non-user group and 294 in the user group. The mean age was 79 years and 77 years in the user and non-user groups, respectively, with a significant number of patients over 80 years (48% user group, 41% non-user group). Male participants were more common in both groups (54% in the user group and 58% in the non-user group). The mean LVEF was 45.7% in the user group and 46.7% in the non-user group. Thirty-five percent of participants in the user's arm had an LVEF of less than 40% versus 39% in the non-user group. The cumulative one-year incidence of the composite of all-cause mortality and heart failure hospitalization was lower in the user group compared to the non-user group (24.5% versus 34.9%, p=0.031; HR 0.62, 95% CI, 0.40-0.96). The cumulative one-year incidence of all-cause death was not significant in the two groups (9.5% versus 10.6%, p=0.715, HR=0.87, 95% CI, 0.41-1.83). The cumulative one-year incidence of hospitalization for heart failure was lower in the user group than in the non-user group (17.7% versus 30.6%, p=0.008; HR=0.51, 95% CI, 0.31-0.84).

Dracup et al. [24] conducted a multi-center randomized controlled study in 12 centers in Kentucky, Nevada, and California to test a counseling and educational intervention for use in rural patients with heart failure with reduced ejection fraction and heart failure with preserved ejection fraction. Participants were randomized to one of the three groups in each recruiting center. Participants in all three groups were given educational material from the American Heart Association and health logs to document phone calls, clinic appointments, emergency department visits, and hospitalizations. Education included information on heart failure, barriers to seeking care, a review of dry weight, symptoms of fluid overload, and messages on the benefits of self-care and diet. The teach-back method was used to ensure understanding. The usual care group participants were 213, PLUS group 198, and LITE group 203. The withdrawal rate from the study was highest in the LITE group and lowest in the control group. The average age was 66 years, and 58.7% of the enrolled participants were male. Fifty-one percent had heart failure with reduced ejection fraction, and 49% had heart failure with preserved ejection fraction. Over the follow-up period, 35% of patients experienced the composite endpoint of cardiac death or hospitalization for heart failure across all groups; the primary outcome was not different across the three groups (p=0.058). There were no differences among the groups regarding hospitalization for heart failure (x2=3.577, p=0.167). Less cardiac mortality was seen in the LITE group (7.5%) compared to the control (17.7%), p=0.003. Differences in cardiac death between the LITE and PLUS groups (p=0.172) and between the PLUS and control groups (p=0.123) were insignificant.

Effectiveness of Self-Management Strategies on Hospitalization for Heart Failure

Boyde et al. [25] carried out a randomized controlled trial at a tertiary health facility in Australia to evaluate the effectiveness of a multimedia educational intervention for patients with heart failure to reduce hospital readmissions. The study recruited 200 randomized patients to the intervention (n=100) and the standard care group (n=100). One hundred and seventy-one participants were analyzed at 12 months. The mean age was similar between the two groups (64 years). More male participants were enrolled in both groups (69% control, 77% intervention). The majority of patients had LVEFs of less than 36% in both groups. The educational intervention reduced the risk of readmission at twelve months by 30% (OR=0.407, 95% CI, 0.216-0.766; RR=0.703, 95% CI, 0.548-0.903). More participants in the intervention group remained event-free compared to the standard care group (59 versus 44, p=0.005). Eleven participants in the intervention group had more than one readmission at 12 months, compared to 27 participants in the standard care group (p=0.004). Chi and Chen [26] carried out a study on daily-based self-management for non-hospitalized patients with heart failure to improve their prognosis. One hundred and seventy-one participants were randomized to receive daily self-management (n=84) or usual care (n=87) and followed up for one year. The self-management intervention involved training and monitoring for signs and symptoms of heart failure. At one year, daily self-management significantly reduced the rate of hospitalization for heart failure (RR=0.41, 95% CI, 0.21-0.79). Further analysis of the intervention group showed reduced all-cause hospitalization and length of hospital stay compared to the standard care group (36.90% versus 81.71%, p<0.05, 16.72 days versus 24.19 days p<0.05).

In China, Cui et al. carried out a randomized controlled trial on a nurse-led structured education program to improve self-management skills and reduce hospital readmissions in patients with chronic heart failure [27]. Two hundred and sixty-five patients with coronary artery disease were admitted, of which 96 participants were randomized to the intervention (n=48) or control group (n=48). The mean age in the intervention group was 55.1 years ±13.4, 56.6 years ± 12.8 for the control group; 68.8% were male in the control group and 72.9 in the intervention group. The mean LVEF was 42.1% ±2.3% in the control group versus 43.5% ± 3 in the intervention group. During the hospital admission, all the participants received guideline-directed medical therapy. The control group received no structured education in the hospital or at discharge. Instead, they received formal education on self-care during hospital stays, done in group sessions with an information pamphlet given at discharge. Table 8 illustrates the summary of interventions in this study.

**Table 8:** Summary of interventions

Education during the hospital stay	First session: one-on-one for 60 minutes; Second session: before discharge from hospital for 60 minutes; Educational material given in both sessions for patients and carers
Follow-up intervention; one-on-one or telephone call	Every four weeks by a specialized nurse for 15-30 minutes. Information on patient instruction concerning medication self-administration and other self-care components was updated.
Outpatient clinics	Review by a physician every eight weeks. Perform physical examination, blood tests, electrocardiogram, and echocardiogram. Reinforcement of education given during telephone calls or one-on-one contact with a specialized nurse.

Readmission was lower in the intervention group compared to the control group (10.4% versus 27.1%, p=0.036). There were no repeat admissions by the end of the study period, and no mortality occurred in either group. Mizukawa et al. [28] conducted an open-label, three-arm randomized controlled study in Hiroshima, Japan. The three arms included the usual care group (UC) (n=19) and the intervention group, divided into two: the self-management group (SM) (n=20) and the collaborative management group (CM) (n=20). The mean age was 74.5 years in the UC, 69.4 years in the SM, and 70.5 years in the CM. Male participants were more prevalent in the UC and SM groups (52.6% and 83.3%, respectively) and balanced in the CM group (50%). The mean LVEF was 42.1% in UC, 42% in SM, and 42.2% in CM. The duration of the study was 24 months, divided into two parts: 12 months of intervention and 12 months of observation. The intervention contents in each group are summarized in Table 9 below.

**Table 9:** Summary of interventions [28]

Intervention	UC	SM	CM
Physicians' visit every two to four weeks; One education session at discharge; Record of weight, blood pressure, and pulse in the self-management book; Six sessions on self-care management once every month; Tele-monitoring by a nurse as needed for 12 months	+ + + - -	+ - + + -	+ - + + +

Rehospitalization for heart failure was noted to be lower in the self-management and collaborative management groups (28.7% and 20.0%, respectively) compared to the usual care group (57.9%), with those randomized to the collaborative group recording the best results (readmission-free survival between the collaborative group and usual care group, p=0.020). This study by Mizukawa et al. was a pilot study that showed that self-management and collaborative management strategies were feasible and beneficial and could be examined further in a larger population.

In a tertiary health facility in Singapore, a three-arm stratified randomized controlled study was undertaken to evaluate the effectiveness of a nurse-led home-based heart failure self-management program (HOM-HEMP) for patients with chronic heart failure [29]. A total of 213 participants were recruited for the study and randomized to the control group, experimental group A, or experimental group B. The mean age was 68.82 years in the control group, 69.08 years in group A, and 66.82 years in group B. Male participants were predominant across all groups (66.1% in control, 69.4% in group A, and 54.4% in group B). Results of this study showed a significant difference in the number of hospitalizations at six-month follow-ups in the experimental versus control groups (p=0.016). The nurse facilitated self-management support for people with heart failure and their family carers (SEMAPHOR) trial was carried out at centers in Birmingham and Darlington, assessing the primary outcome of hospital admission or readmission at twelve months from the time of randomization [30]. Two hundred and sixty patients were randomized to intervention (n=95) and control groups (n=165). The mean age in the intervention group was 70.27 years, compared to 70.79 years in the usual care group. More male participants were recruited in the study, 72.6% in the intervention group versus 72.1% in the usual care group. The primary outcome was admission or readmission to the hospital at 12 months. The secondary outcome was health-related quality of life. The intervention group participants were less likely to get readmitted to the hospital at twelve months, with 19% getting readmitted compared to the 21.2% of the usual care group (RR=0.89, 95% CI, 0.54-1.49, p=0.66), though these results did not reach statistical significance.

Agrinier et al. [31] conducted a prospective, observational cohort study in Lorraine, France, to assess the effectiveness of a disease management program termed Insuffisance CARdiaque en Lorraine (ICALOR) on the incidence of heart failure hospitalization and related healthcare costs. The study included 1223 patients from 19 centers with a median left ventricle ejection fraction of 35% (28-46) who were recruited after the first hospital admission for heart failure. The ICALOR program involved structured education from trained nurses aimed at maintaining and improving adherence to the components of self-care. In the year 2006, when patients were recruited to the study and ICALOR was implemented, the number of hospitalizations for heart failure in Lorraine was 7,489. The expected number was 7,642, based on the increasing trends in France. The ICALOR program was able to avert 183 admissions, translating to 7.19%, with a similar trend observed in 2010 when the follow-up ended. Sezgin et al. [32] conducted a single-center, single-blind, randomized controlled trial at a university hospital in Turkey with the aim of assessing the effectiveness of nursing care and follow-up programs for patients with heart failure on rehospitalization, self-care, and quality of life. Ninety patients were randomized to the intervention (45) and control groups (45). The mean left ventricular ejection fraction did not differ for both groups at baseline (30.22% in the intervention group versus 30.26% in the control group). No significant difference was noted in the incidence of hospitalization at six months between the intervention and control groups (x2=3.85, p=0.05).

The Rehabilitation EnAblement in Chronic Heart Failure (REACH-HFpEF) study was a randomized controlled trial of a facilitated home-based rehabilitation program for patients with preserved left ventricular ejection fraction [33]. It was a single-center, two-group trial carried out in Scotland. Fifty participants were randomized to the intervention group and 50 to the control group. The mean age was 71.8 years in the intervention groups versus 76 years in the control groups. Fewer male participants were available in the intervention (36%) compared to the control group (56%). All participants had a preserved left ventricular ejection fraction of more than 45%. Carer parameters investigated were caregiver burden, quality of life, and contribution to self-care. Hospitalization for heart failure occurred in four participants in the intervention compared to seven in the control groups at six months. Bryant and Himawan conducted a pre- and post-interventional study to investigate the effects of the heart failure self-care program Self-care to Success (HF S2S) on clinical outcomes [34]. Participants were recruited from three outpatient centers in the Midwestern part of the United States of America. Sixty-seven participants were enrolled; 40 completed the study, 20 in the intervention group, and 20 in the control group. The majority of enrolled participants were female (57.5%). The number of hospitalizations in each participant before HF S2S was 0-4, with an average of 0.975 (s=0.947). After the intervention, participant hospitalization ranged from 0-2, with the average dropping down to 0.175 (s=0.446). The decrease in the average number of hospitalizations for heart failure was statistically significant (p<0.001).

Effect of Self-Management Strategies on Healthcare Cost Saving

The Rehabilitation EnAblement in CHronic Heart Failure (REACH-HF) was a two-parallel group, superior, randomized controlled trial undertaken in four centers in the United Kingdom on patients with heart failure with reduced ejection fraction [35]. Usual care was a no-cardiac rehabilitation approach as defined by set guidelines. Two hundred and sixteen participants were recruited and randomized to the intervention (n=107) and control groups (n=109). The mean age in the REACH-HF group was 69.7 years and 69.9 years in the control group. The number of female participants recruited was lower across both groups (24% in the intervention group versus 19% in the control groups). All participants had an LVEF of less than 45%. Overall time input was 8.25 hours per participant, contact time 5.3 hours, and noncontact time 2.9 hours. Adding facilitator training, travel, and consumables, the mean total cost of delivering the REACH-HF intervention was £418.39 per participant, which was deemed affordable and feasible.

A secondary analysis of data from the SEMAPHORE trial (Safety and Efficacy of Tocilizumab vs. Placebo in Polymyalgia Rheumatica With Glucocorticoid Dependence) evaluated the cost-effectiveness of a nurse-led cognitive behavioral self-management program from the perspective of the National Hospital Service compared to usual care by using a cognitive behavior therapy (CBT) manual for patients with heart failure [36]. Ninety-five patients were assigned to the self-management group, and 165 to the usual care group. The mean overall age was 70.6 years. More male participants were recruited (72.1%). The analysis of complete data showed an increase in the cost of intervention by £320.99 compared to usual care and a slight reduction in QALY of 0.021. Further analysis of imputed data did not impact the outcome, with a reduction in QALY persisting (0.004) and an increased cost of intervention of £69.49. The Which Heart Failure Intervention (WHICH?) is the most cost-effective and consumer-friendly in reducing hospital care study was an extended follow-up of a pragmatic multicenter randomized trial cohort evaluating the long-term cost-effectiveness of home versus clinic-based care of heart failure patients in Australia [37]. Two hundred and eighty elderly patients were recruited and randomized to the home-based (n=143) and clinic-based interventions (n=137). The home-based intervention was associated with a greater benefit acquired at a lower cost in terms of QALY, as summarized in Table 10.

**Table 10:** The cost-effectiveness of home-based versus clinic-based interventions for self-management [37]

	Home-based intervention	Clinic-based intervention	p-value
Total health care cost per person (Mean, SD)	$35590±42650	$48691±56747	0.03
Quality-adjusted life years per person (Mean, SD)	2.0±1.3	1.8±1.2	0.078

Reilly et al. undertook an economic evaluation study in North America of a self-care intervention for people with heart failure and diabetes mellitus [38]. One hundred and thirty-four participants were enrolled and randomized to the intervention (n=64) and control groups (n=70). The average age was 57.4 years; more male participants were recruited (65.7%). The average left ventricle ejection fraction was 33.9%. Total health resource use (HRU) and QALYs were reported. The HRU cost per participant was estimated at $9,065 in the intervention group versus $16,712 in the control group. The QALY in the intervention group remained stable from baseline to six months, while a decrease (0.04) was observed in the control group, resulting in a mean QALY difference of 0.04 between the intervention and control groups.

Adherence of Heart Failure Patients to Self-Management Strategies

Several studies in Africa have been done focusing on self-care adherence, most of them emerging from Ethiopia. Dessie et al., 2021 [39] conducted a clustered randomized controlled study in two health facilities on 219 patients in the northwestern part of Ethiopia on the effect of a self-care educational intervention to improve self-care adherence among patients with chronic heart failure. Using the coin flip, one facility was allocated as the intervention facility, and the other was the control facility. The median age in the control group was 50 years, and it was 37.5 years in the intervention group. Fewer male participants were enrolled in the control group (31.6%) compared to the intervention group (52.3%). The primary outcome measure was a heart failure self-adherence score measured by the eight-item Medical Outcomes Study Specific Adherence scale (MOS-SAS). Eight self-care behaviors were assessed. At baseline, there was no difference in the mean self-care adherence scores between the two groups: control 12.1 (±2.8), intervention 12.6 (±2.5). During the second round of health education, mean adherence scores in the intervention group increased compared to the control group: 14.6 (±2.7) versus 20.9 (±7.04). In the third round, the adherence scores remained constant.

Baymot et al. conducted a cross-sectional study on adherence to self-care recommendations and associated factors among adult heart failure patients in public hospitals in Addis Ababa, Ethiopia [40]. Two hundred and ninety-four patients were recruited from five health facilities; 58.6% were male, with 37.5% aged 50 to 69 years. The left ventricle ejection fraction was not specified. Adherence to self-care was measured using the Revised Heart Failure Compliance Questionnaire. Overall, only 32.7% of participants had good adherence to self-care. Adherence to self-care components was as follows: medication 84.7%, weight monitoring 20.1%, low sodium diet 52.7%, fluid restriction 47.3%, regular exercise 57.5%, and appointment keeping 87.4%. Fetensa et al. conducted a hospital-based cross-sectional study on self-care behaviors and associated factors among chronic heart failure patients on follow-up at selected hospitals in Ethiopia [41]. Four hundred and twenty-four participants were enrolled. The mean age was 46.25 years, and 42.9% were male. The left ventricle ejection fraction was not specified, but the majority of the participants were in stage three heart failure (35.4%). Good self-care was defined as patient responses that were less than the mean value of the European heart failure self-care behavior scale; 51.2% of the patients reported good adherence to their self-care recommendations. Urban residence and duration of heart failure of more than one year were positively correlated with good adherence. Seid et al. conducted a hospital-based cross-sectional study on 310 patients in Ethiopia on adherence to self-care recommendations and associated factors among adult patients [42]. The mean age was 49 years, and the majority of participants were female (64.2%). Left ventricle ejection fraction and the clinical stage of heart failure were not specified. The Revised Heart Failure Compliance Scale was used to assess adherence; 22.3% of participants reported overall good adherence to self-care recommendations (95% CI, 17.4%-26.8%). Adherence was best noted for follow-up appointments (85.8%) and medication prescriptions (82.9%). Factors that correlated positively with adherence included male sex (adjusted odds ratio (AOR) 2.34, 95% CI, 1.18-4.62), lack of comorbidities (AOR=2.57, 95% CI, 1.28-5.14), and a good level of knowledge (AOR 2.49, 95% CI, 1.28-4.86).

The Heart of Soweto study evaluated medication adherence, self-care behavior, and knowledge of heart failure in urban South Africa [43]. Two hundred participants were recruited. The mean age was 56 years, and 55% of patients enrolled were male. The mean LVEF was 32%. Adherence to self-care behaviors was defined as per the European Society of Cardiology guidelines. Varied degrees of adherence were reported on the components of self-care behavior, ranging from 2.5 to 98%, as follows: daily weight monitoring at 2.5%, fruit intake at 13%, physical activity at 38%, fluid restriction at 56%, medication adherence at 71%, smoking restriction at 84%, appointment adherence at 95%, and moderate alcohol intake at 98%.

Ding et al. conducted a randomized controlled trial on the effects of telemonitoring on patient compliance with self-management recommendations and outcomes of the innovative telemonitoring enhanced care program for chronic heart failure (ITEC-CHF) in two sites in Australia [44]. One hundred and eighty-four participants were recruited and randomized to intervention (n=91) and control groups (n=93). The overall mean age was 69.5 years; 73% of participants were male. All participants enrolled had a left ventricle ejection fraction of less than 40%. The intervention was associated with a significantly improved compliance adherence score at six months in components of health maintenance (p=0.04), medication adherence (p= 0.05), and diet (p=0.008). In a tertiary health facility in Singapore, a randomized controlled trial on the effect of a nurse-led theory-based program on temporal self-regulation on heart failure self-care was undertaken [45]. One hundred and forty-four participants were recruited and randomized to self-regulation intervention (n=72) or usual care (n=72). At baseline, all participants were noted to have poor self-care as measured by the Self Care for Heart Failure Index (SCHFI) maintenance subscale (52.9±17.2, cut off more than 70). The Patient AcTivated Care at Home (PATCH) randomized controlled trial studied the effects of a home-based activation intervention on self-management adherence and readmission in rural heart failure patients [46]. A hundred patients were recruited and randomized to intervention or usual care groups. Higher self-reported adherence was noted in participants in the intervention group on components of weight checks per week (p=<0.0005), low sodium diet (p=<0.0005) and exercise (p=<0.0005). Janssen-Boyne et al. conducted a randomized controlled trial on the effects of tailored telemonitoring on heart failure patients' knowledge, self-care, self-efficacy, and adherence in a hospital in the Netherlands [47]. Three hundred and eighty-two patients were recruited and randomized to intervention (197) and control groups (185). The mean age of the participants was 71 years in the intervention group and 71.9 years in the control group. 58.4% were male in the intervention group and 60% in the control group. The mean LVEF was 38%, with the majority of patients being below 45% (61%). Daily compliance with the Healthy Buddy telemonitoring intervention was 90% (median 92.3, IQR 84.7-94.9).

Limitations

As with narrative reviews, this study was prone to selection bias in the studies included. Tools used to assess self-management were not uniform across studies. The participants enrolled in the various studies were not ethnically diverse, which limits the generalizability of the review's findings.

## Conclusions

Self-management strategies are effective in reducing mortality and hospitalization for heart failure among patients with heart failure based on studies from other parts of the world, as there is a paucity of data on self-management, mortality, and hospitalization for heart failure in Africa. Most studies on cost-effectiveness agree that self-management reduces the cost of healthcare and has a positive effect on disease-adjusted life years. This cost-effectiveness is driven by reduced hospitalization and reduced length of hospital stay in cases of admission, thus emphasizing the need for the adoption of this strategy in addition to guideline-directed medical therapy. Several studies have been undertaken in Africa on adherence to self-management strategies, especially in Ethiopia. Although some of them are observational, overall adherence is poor to more than 50% of the components of self-management. Randomized controlled studies have evaluated innovative methods of enhancing adherence, such as telemonitoring, the use of mobile telephone applications, and motivational interviewing, that are feasible and cost-effective. Adoption of these innovative methods would improve adherence and subsequently reduce mortality and hospitalization for heart failure.
